# The relative milk production of dairy cattle in tropical Costa Rica that are heterozygous and homozygous for the SLICK1 allele

**DOI:** 10.3168/jdsc.2025-0810

**Published:** 2025-07-16

**Authors:** E.G. Donkersloot, A.M. Winkelman, I.L. Leathwick, J.A. Arias, J. Manuel-Sanchez, R.J. Spelman, S.R. Davis

**Affiliations:** 1 Livestock Improvement Corporation Ltd., Newstead, Hamilton 3240, New Zealand; 2 Centre of eResearch, University of Auckland, Auckland 1142, New Zealand; 3 Genética Chirripó SA, Plaza Rohrmoser, San José 10109, Costa Rica; 4 El Cántaro, Bebedero de Cañas, Guanacaste 50604, Costa Rica

## Abstract

SLICK1 is an allelic variant of the prolactin receptor (*PRLR*) that is found in Senepol beef cattle. The presence of a single copy of this allele produces a short hair coat and confers heat tolerance. We aimed to determine the effect of 2 copies of this allele on milking performance of dairy cattle. Data were obtained from a commercial dairy farm in Costa Rica where crossbreeding of the dairy herd with Senepol had been undertaken for more than 15 yr. Random regression modeling was used to generate lactation curves for individual cattle. Homozygous SLICK1 cows produced 9% more milk (356 L), predominantly over the first 200 d of a 305-d lactation. Milk production of wild-type cattle in the same herd was much lower than SLICK1 carriers, but the number of cows in this group was too low (n = 13) to provide a conclusive assessment of milking performance.

## Highlights

• Cows carrying the SLICK1 allele were milked successfully on a commercial dairy farm.
• Cows homozygous for the SLICK1 allele produced 9% more milk than heterozygotes.
• The extra milk from homozygotes was produced mostly in the first half of lactation.

## Graphical Abstract

**Figure:**
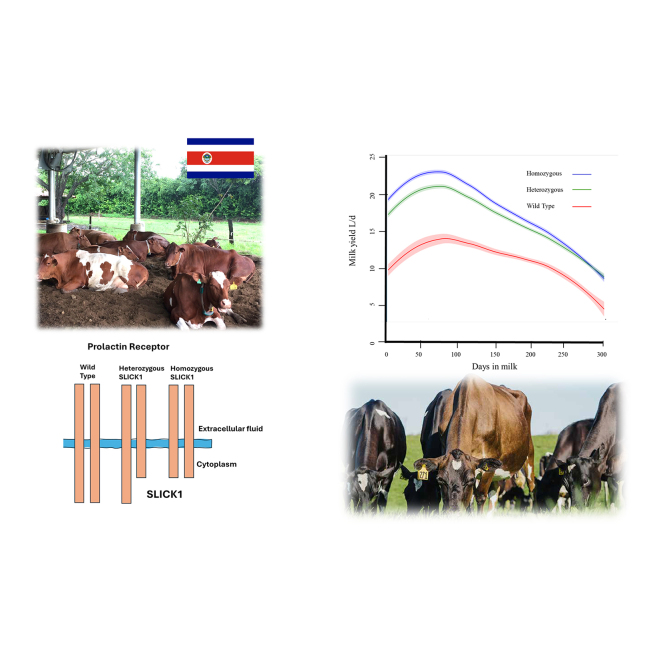
**Summary:** Milk production data from a commercial dairy farm in tropical Costa Rica were analyzed with a randomregression model to compare the lactation curves of cattle that were homozygous (n = 79) or heterozygous (n = 68) for the SLICK1 allele of the prolactin receptor gene, a dominant, genetic variant known to enhance heat tolerance. Homozygous SLICK1 cows produced 9% more milk (356 L), predominantly over the first 200 days of a 305-day lactation, relative to heterozygous cattle. The milk yield of cattle in the same herd, not carrying SLICK1, was much lower than that of SLICK1 carriers, but the number of cows in this group was too low (n = 13) for a conclusive assessment of milking performance to be made.

The Senepol breed of cattle carries a genetic variation that confers heat tolerance (Olson et al., 2003), which has been identified as a single base deletion in exon 10 of the prolactin receptor (*PRLR*) that causes truncation of the cytoplasmic domain of the PRLR protein (Littlejohn et al., 2014). Carriers of the deletion have a characteristically short hair coat (Olson et al., 2003), which led to the genetic variant becoming known as the “slick” gene and the variant from Senepol as SLICK1. Slick is a dominant trait; a single copy of the SLICK1 allele is sufficient to produce the changes in coat characteristics and benefits to heat tolerance (Olson et al., 2003). Several similar truncating mutations have been identified in *PRLR* in other Caribbean beef breeds. All produce a short hair coat and heat tolerance in cattle (Porto-Neto et al., 2018).

Dairy cattle carrying the SLICK1 allele from the Senepol breed have been shown to be more heat tolerant, as assessed by rectal or vaginal temperatures (Dikmen et al., 2014; Donkersloot et al., 2021; Worth et al., 2023) and exhibit greater milk production in summer, relative to non-slick cattle (Dikmen et al., 2014).

However, the milk production of cattle that are homozygous for SLICK1 has not been reported. We identified a commercial farm in tropical Costa Rica where slick dairy cattle had been bred for over 15 yr using Senepol sires across predominantly Jersey and Holstein cows and their F_1_ daughters. This strategy and the data collected presented an opportunity to assess the impact of zygosity for SLICK1 and quantify milk production from farm records in homozygous, heterozygous, and wild-type cows.

All data were obtained from “Hacienda El Cántaro,” a dairy farm in the tropical Cañas-Guanacaste region of Costa Rica (latitude 10°20′07.20ʺ; longitude 85°09′41.32ʺ) at 60 m above sea level. Daytime temperatures rise to a peak of ~38°C in the summer months and ~28°C in the winter months. Night-time temperatures can remain above 22°C in summer. Relative humidity is high, particularly during the rainy season from May to November.

Senepol sires were used to introduce the slick trait into the dairy herd from 2005 onward. Selection of dairy replacements was made on the basis of coat score, with slick-coated calves retained. Pedigree records were kept, allowing identification of breed groups and groups based on proportion of Senepol and dairy breeds (Holstein, Jersey, Ayrshire, Swedish Red).

Through the study period (2014–2017), the herd consisted of 150 to 200 cows of mixed breed and age, milked twice daily. Extensive production and health records were collected from the herd over multiple years and lactations. Following genotyping of the herd for the SLICK1 allele in 2018, data were sourced for the previous 4 seasons for 160 cows that were genotyped for SLICK1. This dataset consisted of a total of 243 lactations.

Cows were milked twice daily at 0300 and 1600 h. Milking was carried out in a herringbone parlor and milk yields were recorded daily from GEA milking control units (M6700, GEA Group AG, Dusseldorf, Germany). Production data were collated using VAMPP dairy management software (version 3.0; https://www.vampp.com).

Cows in the milking herd were fed a proprietary concentrate (VAP, Cooperativa de Productores de Leche Dos Pinos R.L. Alajuela, Costa Rica) at a rate of 1 kg per day per 3 L of milk produced (adjusted weekly), balanced with soyabean flour, a mineral mix, and maize grain to meet nutritional requirements. Roughage was sourced locally from either sugarcane residue (bagasse), pasture (irrigated Brachiaria), or hay, depending upon availability. Cows were held in an open barn during the hottest part of the day (~1000–1500 h), with forced ventilation provided through the barn and premixed feed available ad libitum. Milking cows were treated with bovine somatotropin (Lactotropina; Elanco Animal Health, Indiana) every 2 wk from April 2015 to October 2016. The proportion of cows in each genotype group treated with Lactotropina was similar at ~50% (homozygous, 54%; heterozygous, 50%; wild-type, 46%).

Farm and animal data were collected routinely as part of standard farm practice at El Cántaro. Data included tag numbers, birthdate, age in months, lactation number, sire and dam, breed including Senepol proportion and also liveweight, which was measured in September 2017.

Genotypes for SLICK1 were obtained in 2018 from DNA extracted from tissue samples (ear punch) of animals in the milking herd. SLICK1 genotypes were determined as described by Littlejohn et al. (2014).

Production data (daily volume only) for the period from 2014 to 2017 were combined for a total of 160 cows with a SLICK1 genotype. These animals had one or more calving dates recorded and 68 were heterozygous, 79 were homozygous for SLICK1, and 13 were controls (wild type). In the overall dataset, 93 cows had a single lactation, 52 cows had 2 lactations, 14 cows had 3 lactations, and 1 cow had 4 lactations (a total of 243 lactations).

Following a similar approach to Pereira et al. (2013) for modeling lactation curves with a random regression model, fifth-order Legendre polynomials were used to model the shape of the lactation curves and to generate coefficients describing individual curves. Each lactation curve was standardized to a lactation length of 305 d and adjusted for breed proportions, age, contemporary group, calving month, genotype class, permanent environmental effect, and the random additive genetic effect of the individual animal. The model was fitted using ASREML (Gilmour et al., 2002).

The model used was

$$\begin{array}{c} y_{ijk}=htd_{ij}+age_{i}+HOLprop_{k}+JERprop_{k}+AYRprop_{k}\\ \;+SENprop_{k}+SWRprop_{k}+OTHprop_{k}+\sum_{l=1}^{5}\Phi_{l}\left(d\right)g_{l}\\ +\sum_{l=1}^{5}\Phi_{l}\left(d\right)a_{kl}+\sum_{l=1}^{5}\Phi_{l}\left(d\right)pe_{kl}+e_{ijk}, \end{array}$$

where *y_ijk_* is the test-day observation of milk yield, *i* denotes the lactation (1–4), *j* denotes the herd-year-test-day, *k* denotes the animal; *htd_ij_* is the *j*th herd-year-test-day fixed effect for lactation *i*; *age_i_* is the age at calving in years for lactation *i*; *HOLprop_k_* is the proportion of Holstein breed in animal *k*; *JERprop_k_* is the proportion of Jersey breed in animal *k*; *AYRprop_k_* is the proportion of Ayrshire breed in animal *k*; *SENprop_k_* is the proportion of Senepol breed in animal *k*; *SWRprop_k_* is the proportion of Swedish Red and White breed in animal *k*; *OTHprop_k_* is the proportion of other minor breeds including Santa Gertrudis, Simmental, and Gyr in animal *k*; Φ*_l_*(*d*) is the standardized Legendre polynomial of order *l* calculated at DIM *d* (1–305); *g_l_* is the random genotype effect of order *l*; *a_kl_* is the random additive genetic effect of animal *k* for order *l*; *pe_kl_* is the permanent environmental effect of animal *k* for order *l*; and *e_ijk_* is the random residual associated with record *y_ijk_*.

In matrix notation,

**Y** = **Xβ** + **Z_LP_α** + **Z_LP_pe** + *e*,

where **Y** is a vector containing the milk yield observations; **β** is the vector containing the fixed effects; **X** is an incidence matrix, which indicates for each observation the fixed effects by which it is influenced; **Z_LP_** is an incidence matrix containing the fifth-order Legendre polynomial solutions for each DIM (305 d); **α** is the additive genetic effect matrix containing the standardized fifth-order Legendre polynomials for 305 d; **pe** is the permanent environment animal matrix containing the standardized fifth-order Legendre polynomials for 305 d; and *e* is the residual variance term. Estimates of test day yields for each animal were obtained by multiplying the animal model solution by the Legendre polynomial coefficient, thereby creating values in the same parameter space as the original milk yield data. The permanent environmental solutions and the genotype solutions were also multiplied by the Legendre polynomial coefficient and added to the animal solution to create an adjusted lactation phenotype for each animal. This cleaned phenotype was then scaled by adding the mean of the raw milk yield for the genotype class to create individual, corrected animal yields. Tests of differences among the 3 genotypes on peak milk yield and total production (305 d) were undertaken using the individual animal yields, obtained as described previously.

The estimates of DIM at peak lactation differed slightly by genotype, being d 58, 66, and 71 in homozygous, heterozygous, and wild-type groups, respectively. To facilitate comparisons, the mean solutions and standard errors for peak milk yield were chosen for a peak DIM at 65 d and differences among genotype means tested by Student's *t*-test.

Adjusted production data, by genotype class, are shown in Table 2 and lactation curves for each genotype class are shown in Figure 1. Adjusted total milk production of cows homozygous for SLICK1 was greater than that of heterozygous animals (*P* = 0.007; Table 1), and both slick groups produced substantially more total milk than the wild-type cows (*P* < 0.001). However, the number of lactations in the latter group was small. Most of the difference in production between the SLICK1 homozygotes and heterozygotes was generated in the first 200 d of lactation. There was a significant difference in the adjusted phenotypic peak production (1.4 L/d at DIM 65) between the homozygous and heterozygous SLICK classes (Figure 1; Table 2; *P* < 0.02). Cows in both slick groups produced more milk at peak lactation than cows in the wild-type group (Figure 1; Table 2; *P* < 0.001). Variations in liveweight between genotypes may have contributed to the differences in overall and peak milk production (Table 2). However, as liveweight data were obtained only on a single occasion (September 2017), the true impact of liveweight differences on milk yield cannot be accurately determined.

**Figure 1:**
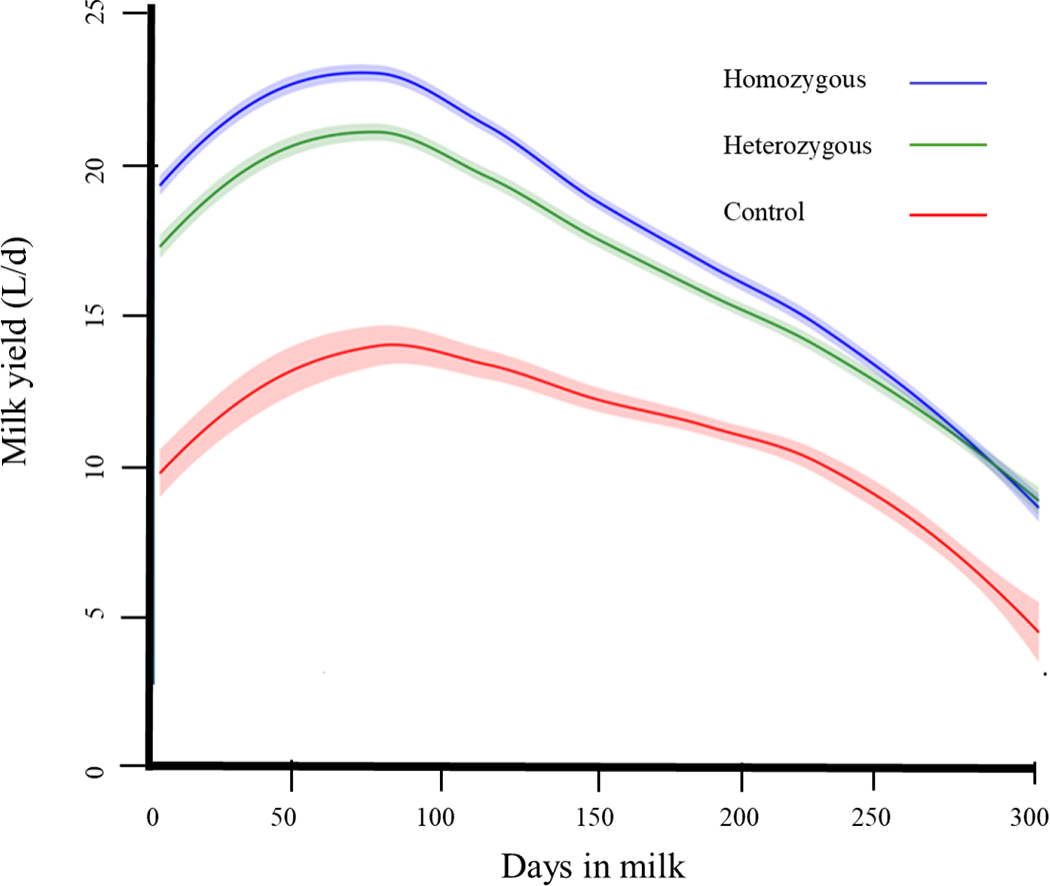
Fitted (Legendre polynomials) lactation curves for homozygous SLICK1 (n = 79), heterozygous (n = 68), and control (wild type; n = 13) cows. The shaded areas show SEM per DIM.

**Table 1:** Herd composition by major breed proportion^1^

Item	Holstein proportion (%)	Jersey proportion (%)	Senepol proportion (%)
Wild type	41	3	31
Heterozygous SLICK	41	4	42
Homozygous SLICK	36	4	46

1 The balance of breeds was provided by Ayrshire, Swedish Red, Montbéliarde, Simmental, and a minor input from several beef breeds.

**Table 2:** Total milk production and peak milk yield by SLICK1 genotype class^1^

Genotype	Homozygous (n = 79)	Heterozygous (n = 68)	Wild type (n = 13)
Total milk production (L)	4,356^a^ (93)	4,000^b^ (92)	2,537^c^ (173)
Peak milk yield (L/d)	22.0^a^ (0.4)	20.6^b^ (0.4)	14.7^c^ (1.0)

a–c Within a row, LSM with different superscripts differ (*P* < 0.05).

1 Numbers in parentheses are the SEM at peak lactation (DIM = 65) or the SEM of the total milk production across the lactation.

Liveweight means per genotype class (SEM in parentheses) were homozygous 527 (9) kg, heterozygous 503 (7) kg, and wild type 486 (19) kg. The only significant difference was between the homozygous and the heterozygous group (*P* = 0.04). The treatments with the lactation stimulant, bovine somatotropin (Lactotropina), may also have introduced some biases into the milk production data, although the number of cows treated was approximately balanced across the genotype groups (as shown previously). The incorporation of the SLICK1 allele into cattle breeding programs provides benefits to both animal welfare and milk production of dairy cattle in hot climates and during summer heat events in temperate or Mediterranean climates. The SLICK1 allele is known to confer heat tolerance on dairy cattle that carry it (Dikmen et al., 2014). The relative advantage for slick cows under thermal load has been reported as a 0.5°C to 0.8°C lower vaginal or rectal temperature (or both; Dikmen et al., 2014; Donkersloot et al., 2021). Furthermore, the respiration rate of slick cattle in hot environments is lower than that seen in wild-type animals (Dikmen et al., 2014). The slick trait has also been associated with higher milk yields in dairy crossbred cattle under tropical or subtropical conditions in Venezuela (Olson et al. 2003) and Puerto Rico (Ortiz-Uriarte et al., 2020), but the impact of zygosity for the SLICK1 allele on milk production of dairy cattle has not been reported previously. Our analysis suggests that there is, on average, a small production advantage for homozygous SLICK1 cows. The small number of animals in the non-SLICK1 class in this report precludes definite conclusions being drawn as to the relative production advantage of slick cows over wild-type cows.

Although the SLICK1 allele may provide increased heat tolerance in cattle in the tropics, it will not fully ameliorate the effects of heat stress. In this tropical environment in Costa Rica, elevations in rectal temperature and respiratory rate were still observed despite slick introgression and management interventions. However, our major conclusion is that homozygosity for SLICK1 is not a disadvantage and may even provide a small advantage for milk production relative to heterozygous animals. Whether this extends to other traits such as health, fertility, and lifetime performance remains to be established.

The mechanism of action of SLICK1 on heat tolerance (and milk production) is not fully understood. The truncation of the cytoplasmic region of the prolactin receptor in SLICK1 carriers could result in stimulation or suppression of prolactin activity through specific intracellular signaling pathways. Suppression of prolactin activity in cattle though genetics (Littlejohn et al., 2014) or fescue toxicosis (Poole et al., 2019) leads to a relatively hairy summer coat, so a slick coat is perhaps suggestive of enhancement of a prolactin signaling pathway or pathways. However, data supporting change in activity of any specific intracellular prolactin signaling pathway in skin are limited. Altman et al. (2025) showed some differences in gene expression that were associated with immune pathways in skin of SLICK1 carriers, including a reduction in hair follicles showing pSTAT3 immunoreactivity.

There is some evidence supporting the view that slick animals have a greater sweating capability compared with non-slick animals (Dikmen et al., 2008, 2014). Increased sweating rates (evaporative water loss) were noted in unclipped areas of skin but not in clipped areas (Dikmen et al., 2008, 2014). The implication of these observations is that the thinner slick coat facilitates cooling by enhancing evaporation of sweat from the skin.

The incorporation of the SLICK1 allele into *Bos taurus* dairy cattle populations from high genetic dairy merit sires will provide an immediate benefit to heat tolerance to the SLICK1 carrier offspring (Carmickle et al., 2022). Whether SLICK1 will produce similar gains in *Bos indicus* or *Bos indicus* × *Box taurus* crossbreds, which already have a short summer coat (Hayman and Nay, 1961), is unknown. Nevertheless, 2 copies of the SLICK1 allele appear to generate a small improvement in lactation performance over a single copy in *Bos taurus* cattle at least in the first 6 to 7 mo of lactation in a tropical environment.
